# MIL-101(Cr), an Efficient Heterogeneous Catalyst for One Pot Synthesis of 2,4,5-tri Substituted Imidazoles under Solvent Free Conditions

**DOI:** 10.3390/nano11040845

**Published:** 2021-03-26

**Authors:** Faranak Manteghi, Fatemeh Zakeri, Owen James Guy, Zari Tehrani

**Affiliations:** 1 Research Laboratory of Inorganic Chemistry and Environment, Department of Chemistry, Iran University of Science and Technology, Narmak, Tehran 1684613114, Iran; f.zakeri2017@gmail.com; 2 Department of Chemistry, College of Science, Swansea University, Singleton Park, Swansea SA2 8PP, UK; O.J.Guy@Swansea.ac.uk; 3 Centre for NanoHealth, College of Engineering, Institute of Life Science-2, Swansea University, Singleton Park, Swansea SA2 8PP, UK

**Keywords:** heterogeneous catalysis, metal-organic frameworks, MIL-101, multicomponent reactions, solvent free

## Abstract

A chromium-containing metal-organic framework (MOF), MIL-101 (Chromium(III) benzene-1,4-dicarboxylate), was used to catalyze the one pot, three component synthesis of some 2,4,5-trisubstituted imidazoles under solvent-free conditions. The advantages of using this heterogeneous catalyst include short reaction time, high yields, easy and quick isolation of catalyst and products, low amount of catalyst needed, and that the addition of solvent, salt, and additives are not needed. This catalyst is highly efficient and can be recovered at least 5 times with a slight loss of efficiency. The structure of the metal-organic frameworks (MOF) was confirmed by X-ray diffraction (XRD) and field emission scanning electron microscopy (FESEM). Fourier transform infrared spectroscopy (FTIR) and proton nuclear magnetic resonance (HNMR) were performed to confirm some of the synthesized products. Experimental data indicated that the optimum amount of catalyst was 5 mg for benzil (1 mmol), 4-chlorobenzaldehyde (1 mmol), and ammonium acetate (2.5 mmol), and the synthetic route to the various imidazoles is performed in 10 min by 95% yield, an acceptable result rivalling those of other catalysts.

## 1. Introduction

Metal-organic frameworks (MOFs), also known as porous co-ordination networks (PCNs), are a class of porous crystalline materials which are constructed from metal ions or metallic clusters as framework nodes connected to rigid bi- or polytopic organic ligands acting as linkers through coordination bonds [1,2,3,4,5]. Varied coordination geometry of metals, bonding preferences of donating atoms, and geometry of bridging ligands can result in polymeric structures with different dimensionalities, high porosity, stability, low densities, uniform pores, and ultra-high surface area. The appropriate choice of metal and organic compound, and possible organization and functionalization of active sites can result in augmented activity and complicated chemo-, regio-, stereo-, shape- and size- selectivity; this makes MOFs attractive, green and eco-friendly candidates with useful applications not only in separation, gas storage, purification, and drug delivery but also in heterogeneous catalysis. Zeolites and other inorganic compounds have been widely used as heterogeneous catalysts. Unlike MOFs, the structure of these siliceous compounds is fixed and unchangeable; besides, their pore size often surpasses 100 A° which renders guest occupancy control quite difficult. On the contrary, MOFs’ crystalline structures can easily be studied by crystallographic techniques which allow us to investigate the distribution of active sites within the framework. MOFs’ structural and dynamic features, such as crystallinity, porosity, and tunability, can be designed to fill the gap between zeolites and surface metal-organic catalysts. These materials cover a much wider range of pore sizes than zeolites; thus, they possess a substantial potential to be used as great heterogeneous catalysts [6,7,8,9].

In homogenous catalysis, metals are used in solution either in the form of metal salts or complexes, being able to catalyze a lot of chemical reactions. However, they suffer from disadvantages such as difficulty in recycling the catalyst or structural decomposition during the reaction. Heterogeneous catalysis overcomes these problems. Metal-organic frameworks can act as heterogeneous catalysts in reactions where the catalytically active sites can be either metal atoms located at the nodes (largely acting as Lewis acids) or any exposed terminal ligands (usually Lewis basic sites). The metal-connecting points in MOFs usually have coordinated small neutral ligands such as water or other solvent molecules that can be simply separated without altering the structure of the framework, generating Lewis acid sites by exposing these metal nodes (activation of the MOF). Moreover, MOFs, due to their heterogeneous nature, can easily be separated from the synthesized products by a simple filtration and be recovered for successive catalytic runs. Thus, the combination of these properties has led to impressive application of metal-organic frameworks in the field of catalysis [10,11,12,13,14].

MOFs have shown superior catalytic performance in various organic reactions such as oxidation, acetylation, epoxidation, hydrogenation, coupling, condensation, alkylation, hydroxylation and cyclization [11], Friedel-Crafts [15], Friedlander [16], Sonogashira [17], Knoevenagel condensation [18], and Biginelli [19], well-known reactions. Chromium(III) terephthalate (MIL-101) with the chemical formula of {Cr_3_(O)(OH)(BDC)_3_(H2O)_2_·nH_2_O} (n~25; benzene-1,4-dicarboxylate (BDC),) and a robust framework is comprised of trimeric chromium(III) octahedral clusters interconnected by BDC molecules, resulting in an augmented MTN (mobil thirty nine topology) zeotype structure. The terminal water molecules can be removed by heating in air or under vacuum, generating two coordinatively unsaturated open metal sites (CUS) per trimeric Cr(III) octahedral cluster producing mild Lewis acid properties. This MOF possesses high thermal, chemical, moisture and solvent stability, and large cavities that enable mass transport via pentagonal and hexagonal windows [20,21].

Imidazole (1,3-diaza-2,4-cyclopentadiene) is a planar five-member ring system with 3C and 2N atoms in 1 and 3 positions. The simplest member of the imidazole family is imidazole itself—a compound with molecular formula of C_3_H_4_N_2_ [22]. The imidazole ring is a constituent of several important natural products including purine, histamine, histidine and nucleic acid. Imidazole derivatives show various pharmacological activities including anti-fungal, anti-bacterial, anti-inflammatory, analgesic, anti-tubercular, anti-depressant, anti-cancer, anti-viral, and anti-leishmanial activity. Medicinal properties of imidazole include it being anticancer, a b-lactamase inhibitor, a 20-HETE (20-Hydroxy-5,8,11,14-eicosatetraenoic acid) synthase inhibitor, a carboxypeptidase inhibitor, a hemeoxygenase inhibitor, an antiaging agent, an anticoagulant, anti-inflammatory, antibacterial, antifungal, antiviral, antitubercular, antidiabetic, and antimalarial [23], as well as a potential inhibitor for the treatment of Alzheimer’s disease [24,25,26]. Thus, several methods to synthesize these heterocyclic scaffolds have been developed. Among these, one-pot syntheses of 2,4,5-trisubstituted imidazoles from 1,2-diketones and aldehydes in the presence of NH_4_OAC is considerably used. Some catalysts can be used in this reaction, such as ytterbium perfluorooctane sulfonate [27], silica sulfuric acid [28], SBA-15/TFE [29], NBS [30], sodium dihydrogen phosphate [31], nano SnCl_4_·SiO_2_ [32], HClO_4_–SiO_2_ [33], TBAB [34], NiCl_2_·6H_2_O [35] and so on. In spite of that, many of these approaches suffer from one or more drawbacks such as a long reaction time, strong acidic conditions, difficult work-up and purification procedures, generation of significant amount of waste materials, occurrence of side reactions, low yields, use of moisture sensitive reagents/catalysts, and excessive use of reagents and catalyst. Therefore, the development of a new mild method to overcome the disadvantages still remains a challenge for organic chemists.

There are a few reports on the application of MOFs as heterogeneous catalysts in one-pot synthesis of multicomponent reactions [36]. Surprisingly, MIL-101 showed great catalytic performance in one-pot, three component synthesis of 2,4,5-trisubstituted imidazoles under solvent free conditions, overcoming the drawbacks mentioned earlier. To the best of our knowledge, this is the first report on the application of MIL-101 as a highly efficient, reusable and green catalyst in the synthesis of substituted imidazoles [37,38,39]. We plan to apply the tri-substituted imidazoles to play a protective role through acetylcholinesterase inhibitory activity in the treatment of Alzheimers’s disease [25].

## 2. Materials and Methods

All chemicals and reagents purchased from Sigma-Aldrich (Dorset, England), and Merck (Merck KGaA, Darmstadt, Germany) were used without further purification. Melting points were measured by an Electrothermal 9100 apparatus (Cole-Palmer, Staffordshire, UK) and are uncorrected. FT-IR spectra were recorded on a Shimadzu FT-IR 8400S spectrometer (Shimadzu, Kyoto, Japan) using the KBr pellet method. HNMR spectra were obtained by an INOVA spectrometer at 500 MHz (Varian, Palo Alto, CA, USA). X-ray powder diffraction (XRD) patterns were recorded using a CuKα radiation source on a BoureVestink, DRON-8 diffraction system. Field Emission scanning electron microscopy (FESEM) was performed on a VEGa/TESCAN, (Kohoutovice, Czech Republic) apparatus.

### 2.1. Synthesis of MIL-101

The MOF was synthesized according to a previously reported method [21]. The details of this synthesis can be found in the supplementary information.

### 2.2. General Procedure for the Preparation of 2,4,5-Trisubstituted Imidazole Derivatives

**Scheme 1:**
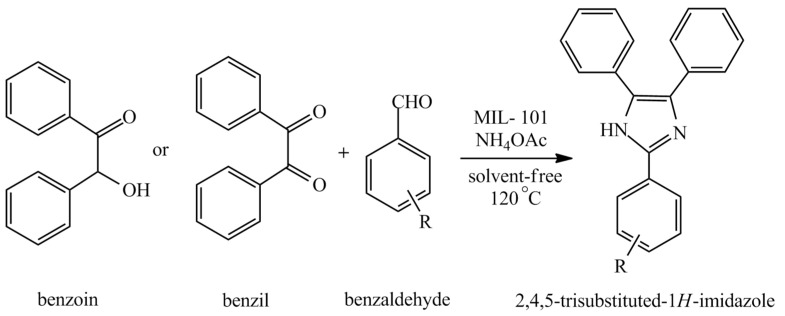
Procedure of synthesizing 2,4,5-trisubstituted imidazole derivatives with starting materials.

In a typical reaction method, the mixture of 1 mmol benzil or benzoin, 1 mmol benzaldehyde, and 2.5 mmol ammonium acetate was heated to 120 °C in the presence of 5 mg MIL-101 as a heterogeneous, solid catalyst under solvent free conditions, as shown in Scheme 1. The reaction progress was monitored by Thin Layer Chromatography (TLC). After the completion of the reaction, the mixture was washed with ethyl acetate and filtered to separate the catalyst. The solution was kept in a refrigerator to give pure crystals of the products in good to high yields, was fully characterized by melting point and spectroscopic methods such as FTIR and H NMR, and has been identified by the comparison of the reported spectral data. The filtrate containing the catalyst was washed several times with ethyl acetate to remove any remaining reaction products and then was heated in a vacuum oven to ensure complete removal of the solvent and reused for successive reactions for at least five times with a slight loss of catalytic activity.

## 3. Results

### 3.1. Characterization of MIL 101

The obtained green powder of MOF was characterized by techniques such as X-ray diffraction (XRD) and field emission scanning electron microscopy (FESEM) indicating that the results were in accordance with previously reported analyses.

**Figure 1:**
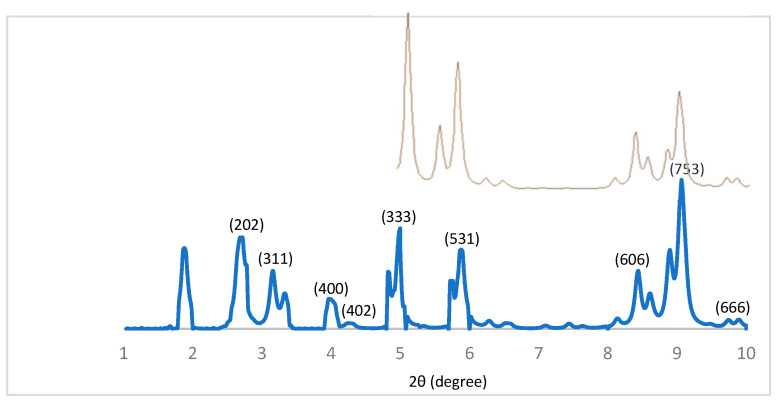
X-ray diffraction pattern of MIL-101(Cr) metal-organic frameworks (MOF) (bottom, blue) and simulated pattern in the range of 2θ = 5–10° (top, brown), with peaks corresponding to crystal planes are indexed.

The XRD (X-ray diffraction) pattern and the simulated pattern of synthesized MIL-101(Cr) are illustrated in Figure 1. The diffraction peaks corresponding to crystal planes are indexed, which match the simulated XRD pattern and/or reported for MIL-101 and thus confirm the formation of this MOF [40,41]. No additional peak was observed, indicating that no other crystalline materials were formed [42,43]. It can be concluded that the sample has a good crystalline structure. The intensive peaks emerged at low angles, proving the high porosity of this material [41].

**Figure 2:**
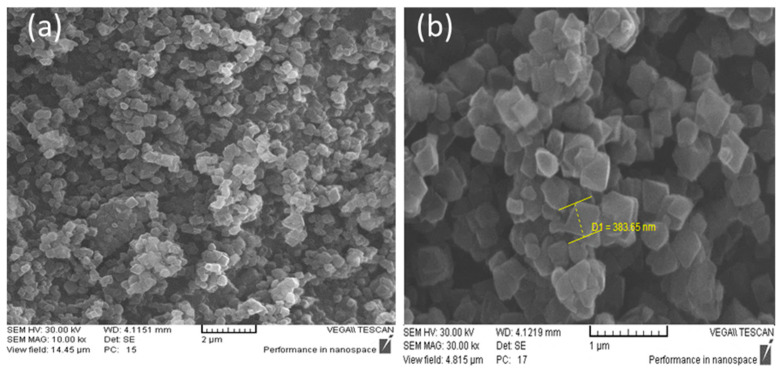
FESEM images of (**a**) MIL-101, (**b**) Zoom on to show sizes of particles indicated in yellow.

The FESEM images of MIL-101 are presented in Figure 2. The particles are octahedral in shape with double-sided pyramid type geometry [41]. These images indicate MIL-101 regular shape and size integrity of the synthesized particles ranging from 200–400 nm [44].

Especially the slightly agglomerated particles shown in Figure 2b, have different sizes, for instance, one is carefully determined as 383.65 nm.

### 3.2. Synthesis of 2,4,5-Trisubstituted Imidazole

Experimental investigations have been conducted on the preparation of trisubstituted imidazole derivatives, showing that parameters such as solvent, temperature, and the amount of catalyst are important factors contributing to the reaction rate and yield. To determine the most appropriate reaction conditions, the reaction was initially carried out under reflux conditions in different solvents and then under solvent free conditions, with the latter proving to be the most efficient. Next, the experiment was conducted at room temperature, without any catalyst, under solvent free conditions. The reaction yield was very low and increased upon introducing the catalyst and raising the temperature to 100, 120 and then 140 °C; 120 °C was the best temperature for the reaction, and raising it further to higher temperatures did not have any substantial effects on the reaction yield nor time. To evaluate the appropriate catalyst amount needed, the model reaction was carried out using different amounts of catalyst (20, 10, 5, 2.5 mg). It was found that the most effective amount of catalyst was 5 mg and larger amounts decreased the yield, while lower amounts did not have any significant effect on the reaction efficiency. The time and temperature of the reaction were also optimized (Table 1).

**Scheme 2:**
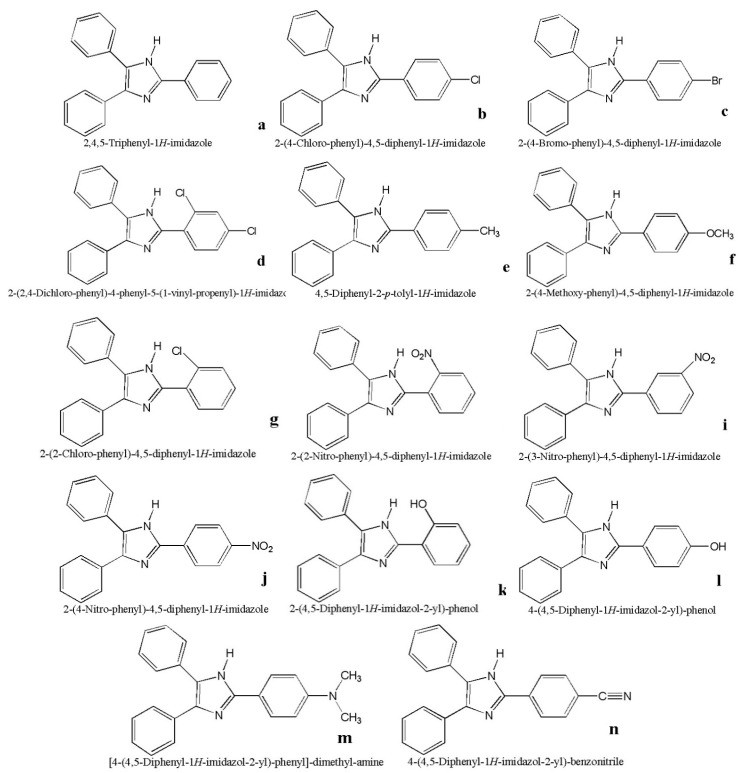
Structures of 2,4,5-trisubstituted 1*H*-imidazoles (**a**–**n**).

To explore the efficiency of the catalyst in the synthesis of 2,4,5-trisubstituted 1*H*-imidazoles **a**–**n** illustrated in Scheme 2, we extended the reaction using different substituted benzaldehydes at 120 °C in solvent free conditions (Table 2). It is evident from Table 2 that when benzoin is used instead of benzil, the reaction time is increased. The electron-deficient aldehyde substituents can stabilize the intermediate in comparison with electron-rich aldehydes. It was found that due to less steric hindrance and the competitive formation of more reactive corresponding imines, electron-withdrawing para substituted groups lead to shorter reaction time and increased catalyst yields rather than electron-donating ortho substituted groups (Table 2).

### 3.3. Selected Data for Some Synthesized Imidazoles

All imidazoles were examined by melting point, and some of them, including b, m, and n, were characterized by FTIR and H NMR spectroscopy (manufacturer, city (State or Province), country); the results were as follows. For 2-(4-Chlorophenyl)-4,5-diphenyl-1*H*-imidazole (b); MP: 259–264 °C, FT-IR (KBr, cm^−1^): 3523 (N–H), 3066 (aromatic C–H), 1487 (C=N), 1579 (C=C). For 4-(4,5-diphenyl-1*H*-imidazol-2-yl)-N,N-dimethylaniline (m); MP: 255–260 °C, FT-IR (KBr, cm^−1^): 3500 (N–H), 3039 (aromatic C–H), 1462 (C=N), 1536 (C=C), peaks at 2800–2900 (aliphatic C–H). Finally, for 4-(4,5-diphenyl-1*H*-imidazole-2-yl)benzonitrile (n); MP: 230–235 °C, FTIR (KBr, cm^−1^): 3400 (N–H), 3055(aromatic C–H), 1440 (C=N), 1602 (C=C), 2223 (C≡N), H NMR (100 MHz, DMSO-*d*_6_): fifteen hydrogen atoms are confirmed by the peak areas. The imidazole hydrogen in 13 ppm is the characteristic peak. The aromatic hydrogens appeared in 7–8.5 ppm with the characterized hydrogen splitting. The peaks with higher chemical shifts which are doubly split are attributed to phenyl group with cyanide substitution, and their hydrogen numbers are confirmed by the peak areas. The mentioned FTIR and H NMR spectra are illustrated in Figures S1–S4.

**Scheme 3:**
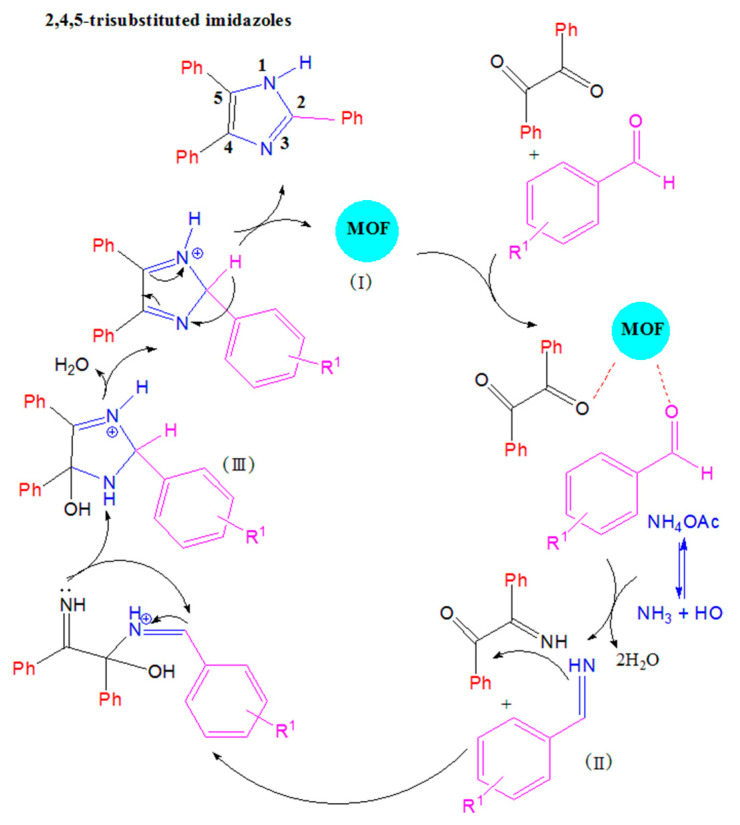
Suggested mechanism for the three-component synthesis of 2,4,5-trisubstituted 1*H*-imidazoles.

A proposed mechanism for the one pot three component synthesis of 2,4,5-trisubstituted 1*H*-imidazoles **a**–**n** is illustrated in Scheme 3. According to the mechanism, ammonium acetate decomposes into ammonia, and acetic acid and the released ammonia is considered as the nitrogen source.

The hydrogen atoms in ammonia and free orbitals of MOF metal centers acting as Lewis acids (I), based on the hard and soft acid and base theory, are responsible for the activation of carbonyl groups. Thus, they can accelerate the rate of imine production through coordination to an oxygen atom from benzaldehyde. Therefore, Cr^3+^ atoms with an unsaturated coordination environment increase the rate of imine formation. The released ammonia from ammonium acetate decomposition attacks the activated carbon on the carbonyl group as a nucleophile. At first, imine intermediate (II) forms, and water molecules release as the side products. Imine intermediate condenses with the carbonyl carbons of 1,2-diketone and then intermediate (III) forms. Finally, by releasing water and after one hydrogen molecule displacement, the imidazole derivative forms.

### 3.4. Catalyst Recycling Procedure

**Figure 3:**
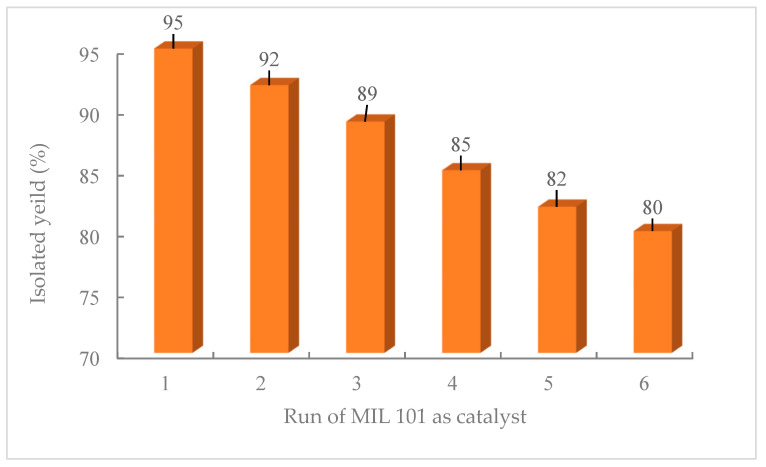
Reusability of MIL-101 MOF as a catalyst for the synthesis of 2,4,5-triphenyl-1*H*-imidazole.

One of the advantages of this MOF as a heterogeneous catalyst is its ability to perform as a recyclable reaction medium. In a typical procedure, after completion of the reaction, the catalyst was washed with ethyl acetate several times to remove any excess product or unreacted precursors and easily separated by simple filtration, then dried. The recycled catalyst could be reused at least five times with a slight loss of catalytic performance. The conversions versus reaction yield of the recycled catalysts are given in Figure 3.

In addition, the turnover number (TON) and turnover frequency (TOF) in the synthesis of all 2,4,5-trisubstituted 1*H*-imidazoles of this work have been calculated and are shown in Table 3. According to the obtained results, TOF has greater values from the TON, because of the short reaction time.

### 3.5. Comparing Catalytic Activity of MIL-101 with Other Catalysts

The catalytic performance of our system was compared with the previously reported results for the synthesis of 2,4,5-trisubstituted-1*H*-imidazole derivatives, and the results are summarized in Table 4. When drawing comparisons with other catalysts, MIL-101 (Cr) exhibits high yield results of 95% and short reaction completion time of 10 min matching some existing systems such as GO-Chitosan and Co/Ni-MOF with the advantage that a much lower amount of MIL-101 is needed to catalyze the reaction. Whilst Yb(OPf)_3_ has a 97% yield, our catalyst offers a combination of additional benefits that Yb(OPf)_3_ and other existing catalysts do not, rendering its overall performance superior; we found that MIL-101 (Cr) showed a 74% lower response time and required 2.7% less to catalyze the reaction. Moreover, the reactions, being conducted using several other catalysts such as Silica Sulfuric acid, Nano SnCl_4_·SiO_2_, TBAB, SBA-15, suffer from several drawbacks such as the use of higher temperatures or solvent, which makes them less environmentally friendly. Finally, the TON and TOF for all the catalysts including our MOF are calculated based on 2,4,5-triphenyl-1*H*-imidazole and reported in Table 4.

## 4. Conclusions

In conclusion, MIL-101 MOF was used as a heterogeneous catalyst for the synthesis of 2,4,5-trisubstituted 1*H*-imidazoles via condensation of 1,2-diketone with various aromatic aldehydes and ammonium acetate under solvent free conditions. The high catalytic activity of the MOF is further highlighted when compared with the other catalysts in this reaction. The results show that some of the reported catalysts suffer from disadvantages such as poor yields, prolonged reaction time, production of waste material, use of hazardous and expensive precursors, harsh reaction conditions, excessive use of catalyst, and use of polar solvents such as ethanol, methanol, acetic acid, water, DMF etc., leading to complex isolation and recovery procedures.

Our system improves on most of previous catalysts drawbacks. Remarkable advantages of this MOF are environmentally friendly reaction conditions, high yields, improve deficiency of reaction using solvent-free systems, reduced reaction times, recyclability of the catalyst, enhanced reaction rates, simplicity of operation, easy work up, and low amount of catalyst needed.

## Figures and Tables

**Table 1:** Optimization of reaction conditions catalyzed by MIL-101.

Entry	Catalyst Weight/mg	Condition	Temperature/°C	Time/min ^a^	Yield/% ^b^
1	_	Solvent free	25	120	0
2	_	Solvent free	120	120	0 ^c^
3	20	Solvent free	100	30	45
4	20	Solvent free	120	10	68
5	10	Solvent free	120	10	80
6	5	Solvent free	120	10	95
7	2.5	Solvent free	120	10	92
8	5	Ethanol, C_2_H_5_OH	reflux	80	80
9	5	Water, H_2_O	reflux	80	35

Experimental condition: benzil (1 mmol), 4-chlorobenzaldehyde (1 mmol), ammonium acetate (2.5 mmol), solvent-free at 120 °C, and 5 mg of catalyst. ^a^ Reaction progress monitored by TLC. ^b^ Isolated yield. ^c^ The entry reported in refs. [30,45].

**Table 2:** Synthesis of 2-aryl-4,5-diphenyl-1*H*-imidazoles (**a**–**n**) using MIL-101 in solvent-free conditions.

Entry	Aryl	Compound	Time/min		Yield/% ^a^		Obtained M.P./°C	Rep. M.P./°C	References
			Benzil	Benzoin	Benzil	Benzoin			
1	C_6_H_5_	**a**	10	15	95	92	270–274	272–274	[46]
2	4-Cl-C_6_H_4_	**b**	10	15	95	87	259–264	260–262	[32]
3	4-Br-C_6_H_4_	**c**	10	18	90	85	257–262	261–263	[47]
4	2,4-Cl_2_-C_6_H_3_	**d**	8	14	95	90	174–176	174–175	[32]
5	4-CH_3_-C_6_H_4_	**e**	10	15	95	92	225–230	228–229	[32]
6	4-CH_3_O-C_6_H_4_	**f**	15	20	92	89	227–228	228–231	[46]
7	2-Cl-C_6_H_4_	**g**	20	25	89	85	186–188	188	[32]
8	2-NO_2_-C_6_H_4_	**h**	15	20	85	85	229–232	230–231	[32]
9	3-NO_2_-C_6_H_4_	**i**	8	14	95	90	300–303	300	[32]
10	4-NO_2_-C_6_H_4_	**j**	10	15	90	87	197–199	194–196	[45]
11	2-OH-C_6_H_4_	**k**	20	15	87	85	201–205	203–205	[47]
12	4-OH-C_6_H_4_	**l**	15	20	92	90	233–234	227–229	[45]
13	4-(CH_3_)_2_N-C_6_H_4_	**m**	15	25	90	85	255–260	255–257	[45]
14	4-CN-C_6_H_4_	**n**	7	15	91	90	230–235	234–236	[45]

^a^ Yields refer to isolated pure products. The known products were characterized and compared by their physical properties with authentic samples.

**Table 3:** Turnover number and turnover frequency of MOF as catalyst.

Entry	Compound	TON	TON	TOF/h^−1^	TOF/h^−1^
		Benzil	Benzoin	Benzil	Benzoin
1	**a**	28	27.2	168.67	108.8
2	**b**	31.2	28.6	188	114.4
3	**c**	33.6	31.8	202.4	106
4	**d**	34.4	32.6	258.64	140
5	**e**	29.4	28.4	177.1	113.6
6	**f**	30	29	120	87.87
7	**g**	29.4	28	89	67.3
8	**h**	28.8	28.8	115.2	87.27
9	**i**	32.2	30.6	242.1	131.33
10	**j**	30.6	29.6	184.33	118.4
11	**k**	27	26.4	81.81	105.6
12	**l**	28.6	28	114.4	84.84
13	**m**	30.2	28.4	120.8	68.26
14	**n**	29	28.8	250	115.2

**Table 4:** Comparison of efficiency of various catalysts with MIL-101 MOF in the synthesis of trisubstituted imidazoles in solvent free conditions.

Entry	Catalyst	Amount /mg	Reaction Condition	Time /min	Yield /%	TON ^a^	TOF ^a^ /h^−1^	Ref.
1	MIL-101	5	Solvent free, 120°C	10	95	28	168.67	Present work
2	GO	15	Solvent free, 120°C	10	75	7.4	44.57	[45]
3	Chitosan	15	Solvent free, 120°C	12	70	6.9	34.5	[45]
4	GO-Chitosan	12	Solvent free, 120°C	10	95	11.7	70.48	[45]
5	NBS	-	Solvent free, 120°C	45	92	-	-	[30]
6	Silica Sulfuric acid	200	Solvent free, 130°C	60	87	0.61	0.74	[28]
7	Co/Ni-MOF	15	Solvent free, 120°C	10	95	8.7	54.38	[36]
8	TBAB	80	Isopropanol, 82°C	20	95	1.7	5.15	[34]
9	Nano SnCl_4_·SiO_2_	30	Solvent free, 130°C	120	95	4.68	2.34	[32]
10	SBA-15	100	TEF, 90°C	180	82	1.36	0.45	[29]
11	Yb(OPf)_3_	6.7	HOAC, C10F18, 80°C	360	97	21.42	214.2	[27]

^a^ TOF and TON were calculated for the synthesis of 2,4,5-triphenyl-1*H*-imidazole.

## Data Availability

The data presented in this study are openly available at https://cronfa.swan.ac.uk/.

## Supplementary Materials

The following are available online at https://www.mdpi.com/article/10.3390/nano11040845/s1, Figure S1: The FTIR spectrum of 2-(4-Chlorophenyl)-4,5-diphenyl-1H-imidazole (b), Figure S2: The FTIR spectrum of 4-(4,5-diphenyl-1*H*-imidazol-2-yl)-benzonitrile (n), Figure S3: The FTIR spectrum of 4-(4,5-diphenyl-1H-imidazol-2-yl)-N,N-dimethylaniline (m),Figure S4: The H NMR spectrum of 4-(4,5-diphenyl-1H-imidazol-2-yl)-N,N-dimethylaniline (m).
