# Supplementary material for: MIL-101(Cr), an Efficient Heterogeneous Catalyst for One Pot Synthesis of 2,4,5-tri Substituted Imidazoles under Solvent Free Conditions
Source: Nanomaterials (Basel). 2021 Mar 26;11(4):845. doi: 10.3390/nano11040845 (PMC8067193; doi:10.3390/nano11040845)
Supplement: Supplementary file 1 [file nanomaterials-11-00845-s001.pdf]

Electronic Supplementary Information

MIL-101(Cr), An Efficient Heterogeneous Catalyst for One Pot Synthesis of 2,4,5-tri Substituted Imidazoles Under Solvent Free Conditions

Faranak Manteghi [1,*,†], Fatemeh Zakeri [1,†], Owen James Guy [2] and Zari Tehrani [3,*]

[1] Research Laboratory of Inorganic Chemistry and Environment, Department of Chemistry, Iran University of Science and Technology, Narmak, Tehran 1684613114, Iran; f_manteghi@iust.ac.ir and f.zakeri2017@gmail.com

[2] Department of Chemistry, College of Science, Swansea University, Singleton Park, Swansea SA2 8PP, UK; O.J.Guy@Swansea.ac.uk

[3] Centre for NanoHealth, College of Engineering, Institute of Life Science-2, Swansea University, Singleton Park, Swansea SA2 8PP, UK; Z.Tehrani@Swansea.ac.uk

* Correspondence: f_manteghi@iust.ac.ir (F.M.); Z.Tehrani@swansea.ac.uk (Z.T.); Tel.: +98-(0)21-73228359 (F.M.); +44-(0)1792-604041 (ZT)

† These authors contributed equally to this work.

Synthesis of MIL-101

The MOF was synthesized according to a previously reported method [21], with blending 2.00 g (5 mmol) $Cr(NO_3)_3{\cdot}9H_2O$ and 0.83 gr (5 mmol) terephthalic acid in 20 ml deionized water, then sonicated and stirred for 30 minutes resulting in a well dispersed, dark blue suspension. The suspension was placed in a Teflon-lined autoclave and kept in the oven at 218 ˚C for 18 hrs. The MOF was separated using a centrifuge and water, methanol, acetone and DMF. The green powder was filtered and dried at 80˚C for 8 h and kept in vacuum oven in 80˚C for 24 h for further activation.

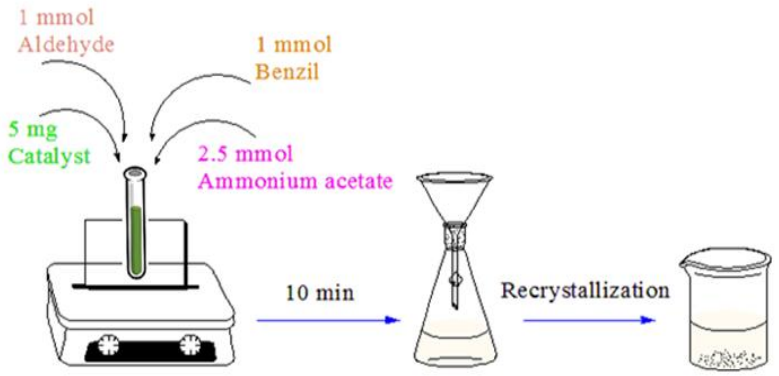

Scheme S1: The synthetis procedure of MIl-101

Some imidazoles including b, n, and m were examined by FTIR and H NMR spectroscopy, the results were as follows.

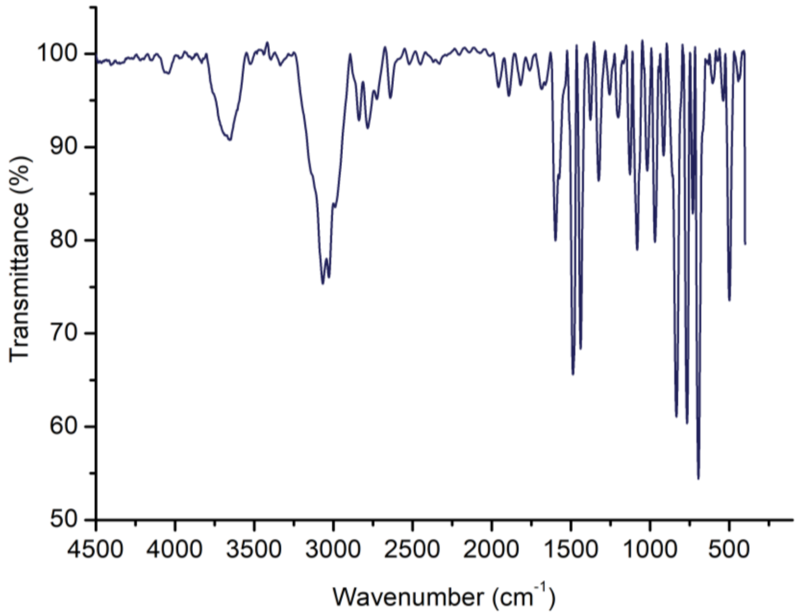

Figure. S1 The FTIR spectrum of 2-(4-Chlorophenyl)-4,5-diphenyl-1H-imidazole (b)

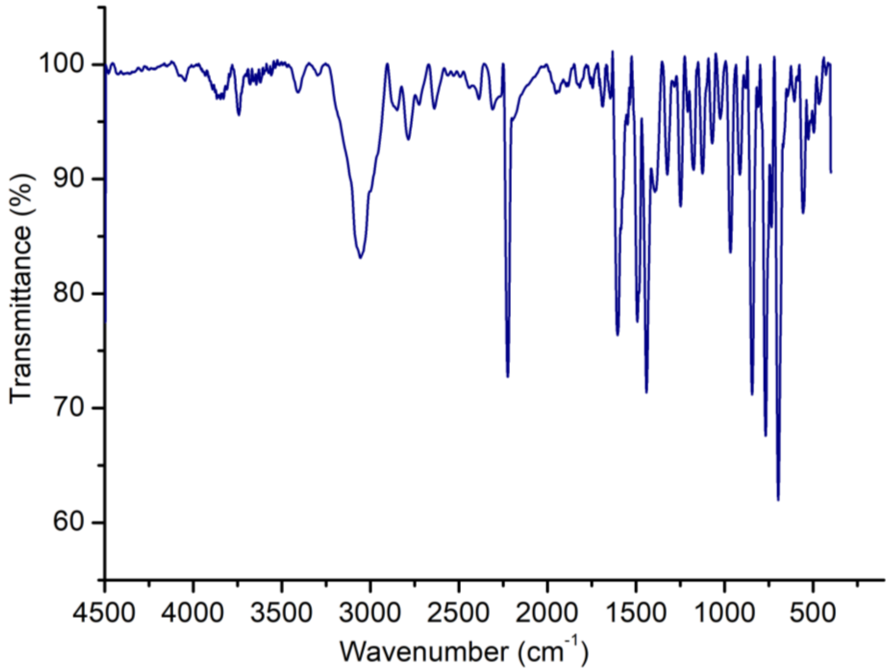

Figure. S2 The FTIR spectrum of 4-(4,5-diphenyl-1*H*-imidazol-2-yl)-benzonitrile (n)

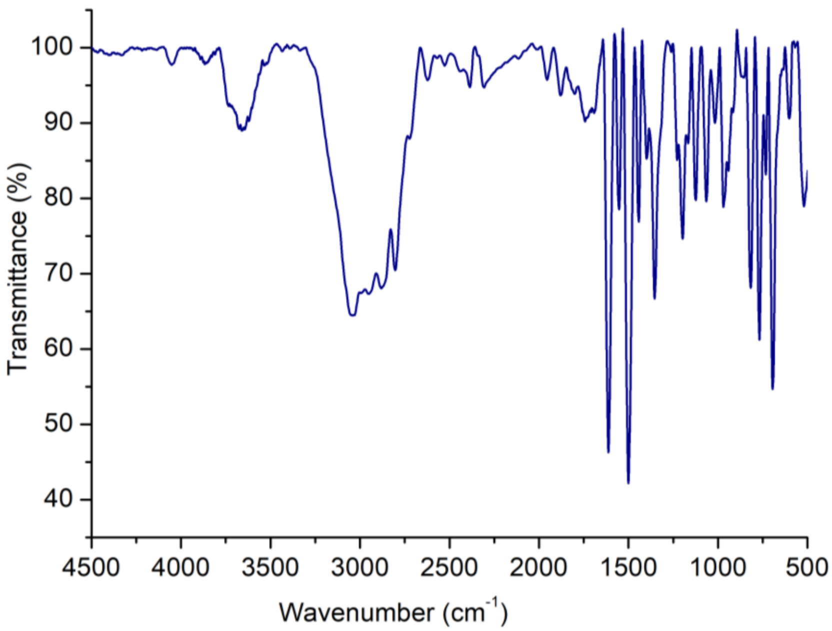

Figure. S3 The FTIR spectrum of 4-(4,5-diphenyl-1H-imidazol-2-yl)-N,N-dimethylaniline (m).

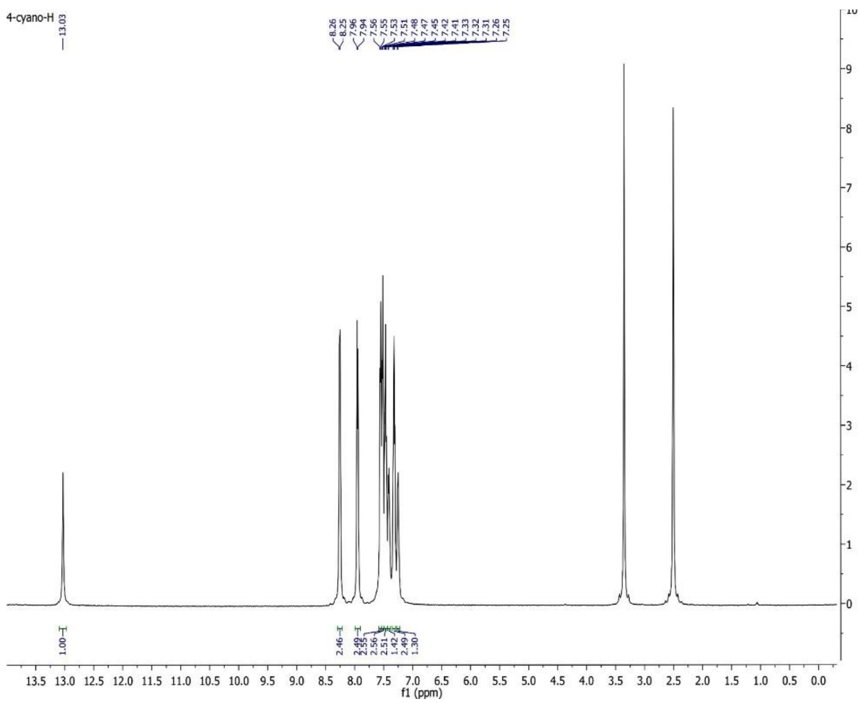

Figure. S4 The H NMR spectrum of 4-(4,5-diphenyl-1H-imidazol-2-yl)-N,N-dimethylaniline (m)
